# Sodium butyrate inhibits colorectal cancer development by reducing M2 macrophage polarization and PD-L1 expression

**DOI:** 10.1128/msystems.00692-25

**Published:** 2025-11-18

**Authors:** Bing Han, Qiong Chai, Qian Chen, Min Liu, Ting Wang, Yu-li Zhang, Zan Li, Zhen Chen, Bo-wang Li, Xian Li, Hua Sui, Qingfeng Tang

**Affiliations:** 1 The Second Clinical Medical College of Henan University of Chinese Medicine, Zhengzhou, China; 2 Nanxiang Branch of Ruijin Hospital, Shanghai Jiao Tong University School of Medicine 56694, Shanghai, China; 3 Gongli Hospital of Shanghai Pudong New Area, Shanghai, China; 4 Henan Provincial Hospital of Traditional Chinese Medicine, Zhengzhou, China, Zhengzhou, China; 5 Jiading Branch of Shanghai General Hospital, Shanghai Jiao Tong University School of Medicine 56694, Shanghai, China; University of Southampton, Southampton, United Kingdom

**Keywords:** colorectal cancer, tumor-associated macrophages, sodium butyrate, NaB, PD-L1, TLR/MyD88 signaling

## Abstract

Short-chain fatty acids (SCFAs), produced by gut bacteria, are being recognized as an important form of anticancer therapy; however, their antitumor potency and underlying mechanisms remain unclear. Here, we used single-cell transcriptomics to identify the mechanism by which the SCFA sodium butyrate (NaB) inhibited the development of colorectal cancer (CRC) and explored new strategies for combining NaB with existing immunotherapies against CRC. An azoxymethane (AOM)/dextran sulfate sodium (DSS)-induced mouse model of colitis and a macrophage-deficient subcutaneous tumor model were used to determine NaB effects on CRC. RNA-sequencing profiled CRC immune landscape changes following NaB treatment. The potential synergy between NaB and programmed death receptor ligand 1 (PD-L1) blockade was explored in macrophage and CRC coculture systems. We showed that NaB markedly reduced inflammation, especially M2 macrophage polarization, tumor burden, histopathological damage, and disease activity index in AOM/DSS mice, and diminished PD-L1^+^ tumor-associated macrophage (TAM) infiltration in CRC tissues. These effects depended on macrophage presence and HDAC/TLR4/MyD88 signaling. The synergy with PD-L1 blockade underscores the need for clinical evaluation of this combination therapy.

**IMPORTANCE:**

CRC remains a leading cause of cancer death worldwide, and new therapeutic approaches are urgently needed. Our study reveals that NaB, a natural gut‐derived metabolite, can reshape the tumor immune environment by limiting pro‐tumor M2 macrophages and reducing PD-L1^+^ macrophage infiltration. By combining single‐cell transcriptomics with mouse models, we pinpoint how butyrate acts through the HDAC/TLR4/MyD88 pathway and demonstrate its synergy with PD-L1 blockade. These findings highlight butyrate’s potential as an accessible, low-toxicity agent to boost existing immunotherapies and offer a clear rationale for clinical trials exploring butyrate–immune checkpoint inhibitor combinations in CRC.

## INTRODUCTION

Colorectal cancer (CRC) is a type of gastrointestinal cancer, which is associated with high morbidity and mortality rates worldwide (1). Immunotherapy, a strategy that redirects the immune system to eradicate tumor cells, has been well tolerated by patients with CRC in the clinic (2); however, response rates have remained suboptimal. This is largely because the interaction between checkpoint molecule programmed cell death protein 1 (PD-1) on T cells and its ligands PD-L1/2 expressed on tumor cells inhibits T cell activation, inducing immune escape and significantly decreasing the efficacy of immunotherapy (3). Immune checkpoint inhibitors (ICIs), which specifically bind to PD-1 or PD-L1, promote T-cell-mediated CRC tumor eradication (4). However, despite the widespread use of anti-PD-1 and anti-PD-L1 antibodies as ICIs in CRC immunotherapy, their effectiveness is limited by the fact that most patients develop primary or acquired resistance during treatment (3, 5). Therefore, identifying reliable adjuvant therapies to fully harness the potential of ICIs represents a promising strategy for halting CRC progression.

Tumor-associated macrophages (TAMs) are important cells in the tumor microenvironment (TME); they play crucial roles in the occurrence, development, and metastasis of CRC (6). Macrophages can differentiate into two phenotypes in response to various stimuli: classically activated macrophages (M1) and alternatively activated macrophages (M2). M1 macrophages, which are induced by factors such as interferon (IFN)-γ and lipopolysaccharide (LPS), mainly play a role in the antitumor response and the clearance of necrotic and apoptotic cells. The majority of TAMs in the TME exhibit the immunosuppressive M2 phenotype, meaning that they promote tumor growth, inhibit immunosurveillance, and facilitate metastasis (7, 8). Additionally, there is a negative correlation between the percentage of CD206^+^ macrophages and CD8^+^ T cells in CRC tissues, indicating that CD206^+^ macrophages inhibit the infiltration of CD8^+^ T cells into the TME, thereby inducing immune escape (9). These findings highlight M2 macrophages as a crucial target for antitumor immunity.

Short-chain fatty acids (SCFAs), which include acetate, propionate, and sodium butyrate (NaB), are produced through the fermentation of dietary fiber by gut bacteria (10). Among these, NaB has demonstrated protective effects against intestinal tumorigenesis. NaB has been shown to enhance the mitochondrial function of colon epithelial cells to increase their energy supply and decrease autophagy, as well as maintain the permeability, density, and homeostasis of intestinal epithelial cells (11). Additionally, NaB reduces the production of inflammatory factors in favor of tumor clearance (12, 13). Previous studies have shown that NaB acts as a histone deacetylase inhibitor (HDACi); thus, NaB can inhibit tumor progression by inducing the cell cycle arrest, apoptosis, and autophagy of tumor cells by reducing their HDAC levels (14, 15). NaB also induces CD4^+^ T cells to produce interleukin (IL)−22, thereby enhancing intestinal immune function by inhibiting HDAC and G protein-coupled receptor 41 activity (16). Furthermore, NaB increases c-Fos expression, which in turn suppresses cystine/glutamate transporter expression, thereby increasing the sensitivity of CRC cells to ferroptosis (17). NaB also induces granzyme B^+^, IFN-γ^+^, and TNF-α^+^CD8^+^ T cells, which inhibit tumor growth and enhance the efficacy of anti-PD-1 therapy (18). Moreover, NaB inhibits CRC cell proliferation by activating the adenosine-monophosphate-activated protein kinase signaling pathway, which causes CRC cells to arrest in the G2/M phase, while reducing their mitochondrial membrane potential, resulting in reactive oxygen species production (19). Finally, NaB improves the intestinal microenvironment by increasing the relative abundance of beneficial gut bacteria, reducing CRC progression (20), and enhancing the effects of anti-PD-1 and anti-PD-L1 therapies against CRC (21).

These studies have shown that NaB is a promising anticancer agent, which could be used alongside other therapies such as ICIs. In this study, we used both *in vivo* and *in vitro* methods to evaluate the effect of NaB on the growth of CRC tumors, focusing specifically on macrophage polarization and related mechanisms. Our research highlights the potential of NaB to enhance the efficacy of CRC immunotherapy by inhibiting M2 macrophage polarization and reducing PD-L1 expression.

## MATERIALS AND METHODS 

### Mouse model of experimental colitis

**Fig 1:**
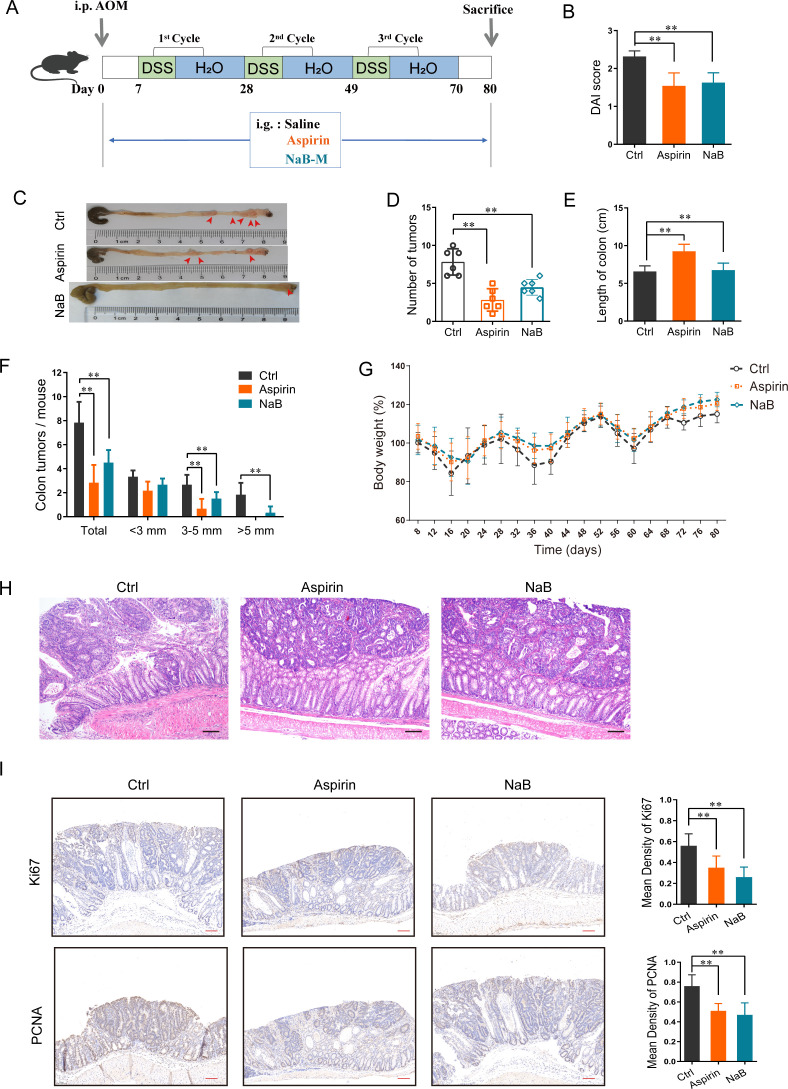
Sodium butyrate (NaB) reduces colorectal carcinogenesis in AOM-/DSS-induced mice. (**A**) Experimental design and timeline for AOM-/DSS-induced mice treated with saline, aspirin, or NaB-M (*n* = 8 per group). (**B**) During the experiment, the DAI score of mice was assessed with body weights, stool, and body posture daily. (**C**) Representative colonic morphologies of the CAC model mice with different treatments. (**D, E**) Effects of NaB on the number of tumors (**D**) and colon length (**E**) in the CAC mouse model. (**F**) The tumor size distribution in the intestine was listed and compared with the control. (**G**) Body weight of mice was measured and recorded every 4 days during the development of colorectal carcinogenesis. (**H**) Representative hematoxylin and eosin (H&E) staining images of different group mice. Scale bar = 50 µm (**I**) Immunohistochemical staining with an antibody against Ki67 and PCNA in the colon of different groups of mice (left panel), scale bar = 100 µm. Bar graphs showing the mean percentages of Ki67 and PCNA-positive area (right panel). The data are presented as the mean ± SD (*n* = 5), with Welch’s correction, two-tailed *t*-test. **P < 0.01.

Specific-pathogen-free C57BL/6J male mice (23 ± 2 g, 6–8 weeks old) were randomly divided into three groups: the NaB group, the aspirin group, and the control group (Fig. 1A). To induce CRC, mice were given a single intraperitoneal injection of azoxymethane (AOM) (10 mg/kg body weight), as previously described (22). A week after the AOM injection, the mice were given 2% dextran sodium sulfate (DSS) (wt/vol) in their drinking water for 7 days. On Day 14, mice were given standard drinking water for 2 weeks. The abovementioned DSS and water steps were repeated on Days 28 and 49 as the second and third cycles of DSS administration. NaB was administered daily at a concentration of 100 mg/mL (23). Aspirin (30 mg/kg) served as the positive control; it was selected as the positive control because of its well-documented efficacy in inhibiting the growth of CRC cells (24). Phosphate-buffered saline (PBS) was used as the vehicle control throughout the entire experimental cycle. Mice were sacrificed on Day 80, and samples were prepared for assessment. All animals were housed under 12 hour light/12 hour dark cycle conditions, and all mouse experiments were performed in accordance with the Chinese Experimental Animals Administration Legislation.

### Assessment of general conditions

During the experiment, the body weight, defecation characteristics, and mental status of the mice were recorded, and fecal occult blood was tested. Disease activity index (DAI) scores were determined according to previous studies (25).

### Hematoxylin and eosin (H&E) staining

After the mice were sacrificed, their whole intestines were immediately removed, washed with ice-cold PBS, and opened longitudinally, as previously described (26). The tissue sections were fixed in 10% formalin, embedded in paraffin, and stained with H&E for pathological evaluation by a pathologist blinded to the experimental groups. Histopathologic findings and the degree of dysplasia were assessed according to the histopathological grading criteria for colorectal adenomas (27) (Table S1).

### Single-cell RNA sequencing (scRNA-seq)

mRNA was purified from total RNA using poly-T oligonucleotides attached to magnetic beads. Subsequently, a single-cell RNA sequencing library was prepared using version 1 of the Chromium Single Cell 3 Library of the Gel Bead & Multiplex Kit (10 x Genomics, Pleasanton, CA, USA). Sequencing was carried out using the Illumina NextSeq500 platform (NovelBio Co., Ltd., Shanghai, China). Prior to downstream analysis, cells were filtered by the unique molecular identifier (UMI) (<100,000 UMIs), gene number (<6,000 genes), and mitochondrial gene percentage (“percentage. MT” <10%). Cell Ranger (version 3.0.1) with default parameters was used for scRNA-seq data analysis, which encompassed sample multiplexing, barcode processing, and the quantification of unique molecular indices for single-cell gene expression.

### Subcutaneous CRC tumor implantation

The subcutaneous tumor model was established in C57BL/6 J mice using MC38 cells, as previously described (28, 29). A single control group was used for Fig. 4B and 5C due to identical modeling procedures. When the tumor volume reached ~100 mm^3^, the mice (*n* = 5) were randomly treated with PBS, NaB, or anti-PD-L1 antibody (BioXcell, West Lebanon, NH, USA, Cat#BE0101). On the basis of a previous study (30), two oral doses of NaB were used: low (NaB-L), 50 mg/kg; and high (NaB-H), 200 mg/kg. αPD-L1 monotherapy (100 μg per mouse, once every 3 days) was administered to the mice in the αPD-L1 group (31). Subsequently, tumor sizes (volume = [length × width^2^]/2) and mouse weights were recorded every 3 days. The experiment was terminated when the tumor volume was >2,000 mm^3^ or when the mouse died. Finally, the tumor volume, tumor weight, and mouse body weight were measured, and tumor tissues were stored in –80°C or fixed in 10% formalin for downstream analysis. Mouse tumor volumes were recorded until all mice reached the experimental endpoint.

### Colony formation assay

Caco-2 cells (1 × 10^3^) in 2 mL of complete medium were added to each well of a six-well plate, shaken to mix, and cultured in a cell incubator for approximately 10 days. The cells were fixed with 4% paraformaldehyde, stained with 1 mL of 0.5% crystal violet for 10 min, rinsed with tap water, dried, and photographed as described previously (26).

### Invasion and migration assays

Raw264.7 cells, THP-1 cells, and bone-marrow-derived macrophages (BMDMs) were used in the Transwell assays. Transwell assays assessing cell migration or invasion were performed on 24-well plates with inserts (BD Biosciences) according to the manufacturer’s instructions. Macrophages were seeded in the bottom chamber at a concentration of 5 × 10^5^ cells/well, while the CRC cells were seeded at 3 × 10^5^ cells/well in the upper chamber and were allowed to migrate or invade for 12–24 h before fixation for crystal violet staining. NaB was added to the macrophage culture medium; the macrophages were harvested after 24 h. Then, the cells on the upper surface of the polycarbonate membrane were removed with cotton swabs, while the cells on the lower side were fixed with 100% methanol, stained with 0.05% crystal violet, and imaged under a microscope. The number of migrating cells was quantified from three independent experiments.

### Immunohistochemistry

Colon sections were examined by immunohistochemistry after staining with anti-Ki-67 (Cell Signaling Technology, Danvers, MA, Cat#12202, 1:400) and anti-PCNA (Cell Signaling Technology, Danvers, MA, Cat#2586S 1:400) antibodies, as previously reported (32). The Ki-67 index was quantified using ImageJ software from 3–5 randomly selected microscopic fields per sample. The DeadEnd Fluorometric TUNEL System (Promega) was used to perform the TUNEL assay. The average number of TUNEL-positive cells from 3–5 random fields per tumor was determined by three researchers blinded to the nature of the samples.

### Flow cytometry

BMDMs were harvested from mice. The cells were incubated with anti-mouse antibodies targeting the following molecules at 4°C in the dark for 30 min: CD86 (Alexa Fluor 488, Invitrogen, Lot#2473673), CD206 (PE, eBioscience, 12-2061-82), F4/80 (APC, eBioscience, 17-4801-82), CD11b (APC, eBioscience, Cat#101202), CD4 (PE/Cynine7, Biolegend, Cat#100421), CD8 (APC, BD Biosciences, 553035), PD-1 (PE, BD Biosciences, Cat#551892), PD-L1 (PE, Biolegend, Cat#124307), CD45 (FITC, Invitrogen, 2312013), and CD3 (Alexa Fluor 700, Biolegend, Cat#100215). The cells were washed twice with PBS and resuspended in 500 µL of PBS. Monoclonal antibodies with irrelevant specificities conjugated to Alexa Fluor 488, PE/Cyanine7, Alexa Fluor 700, APC, FITC, or PE- were used as negative controls. The light scatter characteristics of each sample (10^5^ cells) were analyzed using flow cytometry (CytoFLEX LX, USA).

### Enzyme-linked immunosorbent assay (ELISA)

Distal colon tissues were homogenized to extract total protein, as previously described (33). The protein concentration was measured using a BCA kit (Beyotime, Beijing, China). The levels of the inflammatory cytokines/chemokines IL-6, IL-1β, TNF-α, CCL2, and CXCL4 were measured using the relevant ELISA kits according to the manufacturer’s instructions (Dogesce, Beijing, China; Andygene Biotechnology Co., Ltd, Beijing, China). A microplate reader (Gibco; Thermo Fisher Scientific, Waltham, MA, USA) was used to measure the absorbance at 450 nm.

### Macrophage and CRC cell coculture system

THP-1 cells were seeded in the upper chambers (0.4 µm pore size membrane) of a Transwell plate at 4 × 10^5^ cells/well and primed with 100 ng/mL PMA for 48 h. The resulting macrophages were then treated with an anti-PD-L1 antibody (Cat#758206, BioLegend, San Diego, CA, USA) for 24 h. CRC cells were plated at 2 × 10^6^ cells/well in the lower chamber of a six-well Transwell plate (Corning, USA). Then, NaB (1.6 mM) was added into the culture medium for 24 h, and the culture supernatant and macrophages were collected for further experiments.

### Western blotting

CRC-conditioned medium (CRC-CM) was prepared from Caco-2 cells, as previously described (34). THP-1 cells and BMDMs were pretreated with CRC-CM for 24 h and then with NaB (0.4-1.6 mM) for a further 24 h. After that, the cells were lysed with a lysis buffer (Beyotime Inst. Biotech, Beijing, China), and total protein was extracted. Protein concentrations in the supernatants were determined using a bicinchoninic acid protein assay kit (Beyotime Inst. Biotech, Beijing, China); 30 mg of protein was resolved by 10% sodium dodecyl sulfate-polyacrylamide gel electrophoresis. After blocking with 5% non-fat milk in Tris-buffered saline containing 0.1% Tween-20 (TBS-T) for 1 h, membranes were washed five times with TBS-T. The membranes were then incubated overnight at 4°C with primary antibodies against HDAC1 (1:2,000), TLR4 (1:2,000), MyD88 (1:2,000), and GAPDH (1:1,000) (all from Cell Signaling Technology, Danvers, MA, USA). Next, the membranes were washed five times with TBS-T (10 min per wash) and then incubated with horseradish peroxidase-conjugated secondary antibodies (goat anti-rabbit, 1:1,000; rabbit anti-goat, 1:1,000; both from Beyotime Inst. Biotech, Beijing, China) for 1 h at room temperature. After the final five washes with TBS-T, the membranes were visualized using an enhanced chemiluminescence kit (ECL; Applygen Inst. Biotech, Beijing, China).

### RNA extraction and qPCR analysis

RNA was extracted from CRC cells and TAMs using the Tripure Isolation reagent (Roche), as previously described (35). Complementary DNA (cDNA) was synthesized at 42°C for 15 min, followed by 95°C for 30 s, using the 4 × Reverse Transcription Master Mix (EZBioscience). Subsequently, quantitative mRNA analysis was performed using SYBR Green (Yeasen, China). Quantitative real-time PCR (qRT-PCR) for all analyzed genes was carried out using the PrimeScript RT reagent Kit (Takara Biotechnology); qPCR was performed using the PrimeScript Reagent Kit Perfect Real-Time (Takara Biotechnology). Relative gene expression was calculated using the 2^−ΔΔCt^ method, where ΔCT = CT (target gene) − CT (housekeeping gene), and Δ(ΔCT) = ΔCT (treated) − ΔCT (control). *GAPDH* was used as the housekeeping gene. The primer sequences are provided in Table S2.

### Molecular dynamics simulation

Three-dimensional protein structures were retrieved from UniProt (https://www.uniprot.org/) in SDF format and imported into AutoDockTools 1.5.6 for preprocessing, including removal of water molecules and non-essential ligands. The prepared structures were saved in PDBQT format and subjected to molecular docking simulations using AutoDock Vina. The lowest-energy binding conformations were identified and exported as PDB files using Discovery Studio 4.5. Docking poses were visualized and analyzed with PyMOL.

### Statistical analysis

The data were expressed as the mean ± standard error of the mean (SEM) and were analyzed using one-way analysis of variance (ANOVA). In all cases, an F-ratio with a *P*-value < 0.05 indicated statistical significance; there was no significant variance inhomogeneity. *Post hoc* comparisons were conducted using the Newman–Keuls test or a *t*-test, as appropriate. A *P*-value < 0.05 was considered a measure of statistical significance. Mouse body weight changes were analyzed using two-way ANOVA, followed by the Newman–Keuls multiple comparison test, with treatment and time set as variables. Statistical analyses were performed using GraphPad Prism 9.0 (GraphPad Software, La Jolla, CA, USA).

## RESULTS

### NaB inhibits AOM/DSS-induced intestinal tumorigenesis

To investigate the effect of NaB on CRC tumorigenesis, C57BL/6 J mice were treated with AOM and DSS to induce CRC and then were orally administered PBS, aspirin, or NaB. We chose NaB-M (100 mg/kg) as the intervention dose for our experiments, in accordance with a previous study, due to its favorable safety profile (36). On Day 80, the AOM/DSS mice were sacrificed, and their intestinal tumor samples were collected (Fig. 1A). We found that the disease activity index (DAI) and the number of tumors per mouse were significantly lower in the mice treated with NaB or aspirin than in those receiving saline (Fig. 1B through D). NaB and aspirin treatment also increased colon length (Fig. 1E), reduced the number of tumors measuring 3–5 mm and >5 mm (Fig. 1F), and increased mouse body weight relative to the control (Fig. 1G). Moreover, NaB and aspirin treatment lowered the extent of inflammation, reduced tumor heterogeneity (Fig. 1H; Table S2), and decreased the number of tumor cells expressing Ki67 and PCNA (both markers of cell proliferation) (Fig. 1I). These findings suggest that NaB, like aspirin, has an inhibitory effect on the occurrence of CRC.

### scRNA-seq reveals heterogeneity of TAMs and CD8^+^ T cells in the TME of CRC model mice

**Fig 2:**
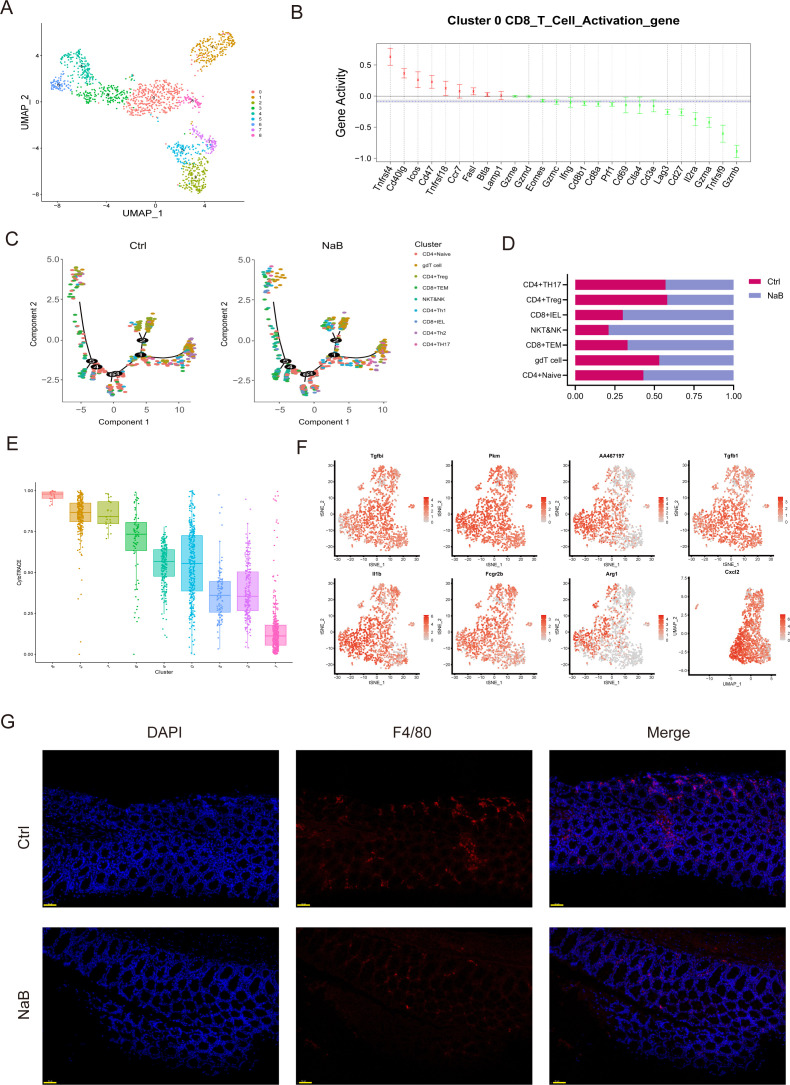
scRNA analysis reveals the involvement of PD-L1 in NaB-mediated macrophage differentiation. (**A**) UMAP plot of T cells showing annotated clusters in vehicle-treated and NaB-treated CAC model mice. (**B**) Expression levels of selected known hallmark genes among various types of CD8^+^ T cell. (**C**) Pseudo-time trajectory analysis of T cells based on groups and subtypes and faceted plots showing cell types in pseudo-time series. (**D**) Bar plot showing a comparison of the proportions of the main cell types between Ctrl and NaB groups. (**E**) CytoTRACE.boxPlot of macrophages. (**F**) Expression levels of selected known hallmark genes among various types of macrophages. (**G**) Immunofluorescence micrographs of 40,6-diamidino-2-phenylindole (DAPI) (blue) and F4/80 (red) expression. Scale bar = 50 µm

To gain a deeper understanding of how NaB inhibits CRC development, we performed scRNA-seq analysis of immune cells sorted by flow cytometry from the colon tissues of either control or NaB-treated mice. Uniform Manifold Approximation and Projection (UMAP) identified 13 clusters of cells in an unbiased manner (Fig. 2A). Analysis of cluster percentile distribution revealed that clusters 1 and 8 were particularly enriched in the NaB-treated mice, whereas cluster 8 was enriched in CD8^+^ T cells (Fig. 2A). Importantly, gene ontology analysis of biological processes revealed that cluster 1 was enriched in genes involved in the production of the anti-inflammatory cytokine IL-10, as well as in tolerance induction and defense responses to bacteria (Fig. 2B). The results of our cell-trajectory analysis showed that T cells in the NaB group tended to polarize toward CD8^+^ T cells (Fig. 2C). Compared to the control group, the NaB group exhibited a significant increase in the numbers of both CD8^+^ intraepithelial lymphocytes (IELs) and CD8^+^ effector memory T cells (TEMs) (Fig. 2D). Transcriptomic profiling revealed distinct cellular subpopulations: Cluster 1 was characterized by PD-L1-expressing CD8^+^ T cells, while Cluster 8 predominantly comprised Arg-1^+^ CXCL2^+^ macrophages (Fig. 2E), suggesting divergent functional states within the immune microenvironment. The identified transcripts were associated with the polarization of macrophages, including that of the M2 subtype (Arg-1 and CXCL2), transcription (PKM and fcgr2b), and the inflammatory response (TGF-β and IL-1β) (Fig. 2F). Further, immunofluorescence analysis demonstrated abundant F4/80^+^ cells in the control group, indicating high macrophage activity. In contrast, the NaB group exhibited reduced F4/80 expression, diminished fluorescence intensity, and sparse distribution of positive cells, suggesting that NaB significantly suppresses intestinal macrophage activity in mouse models of CRA. Overall, the transcriptomic signature identified had the typical features of tolerogenic PD-L1-expressing CD8^+^ T cells and M2 macrophages.

### NaB inhibited M2 polarized macrophage activity through the HDAC/TLR4/MyD88 signal pathway

**Fig 3:**
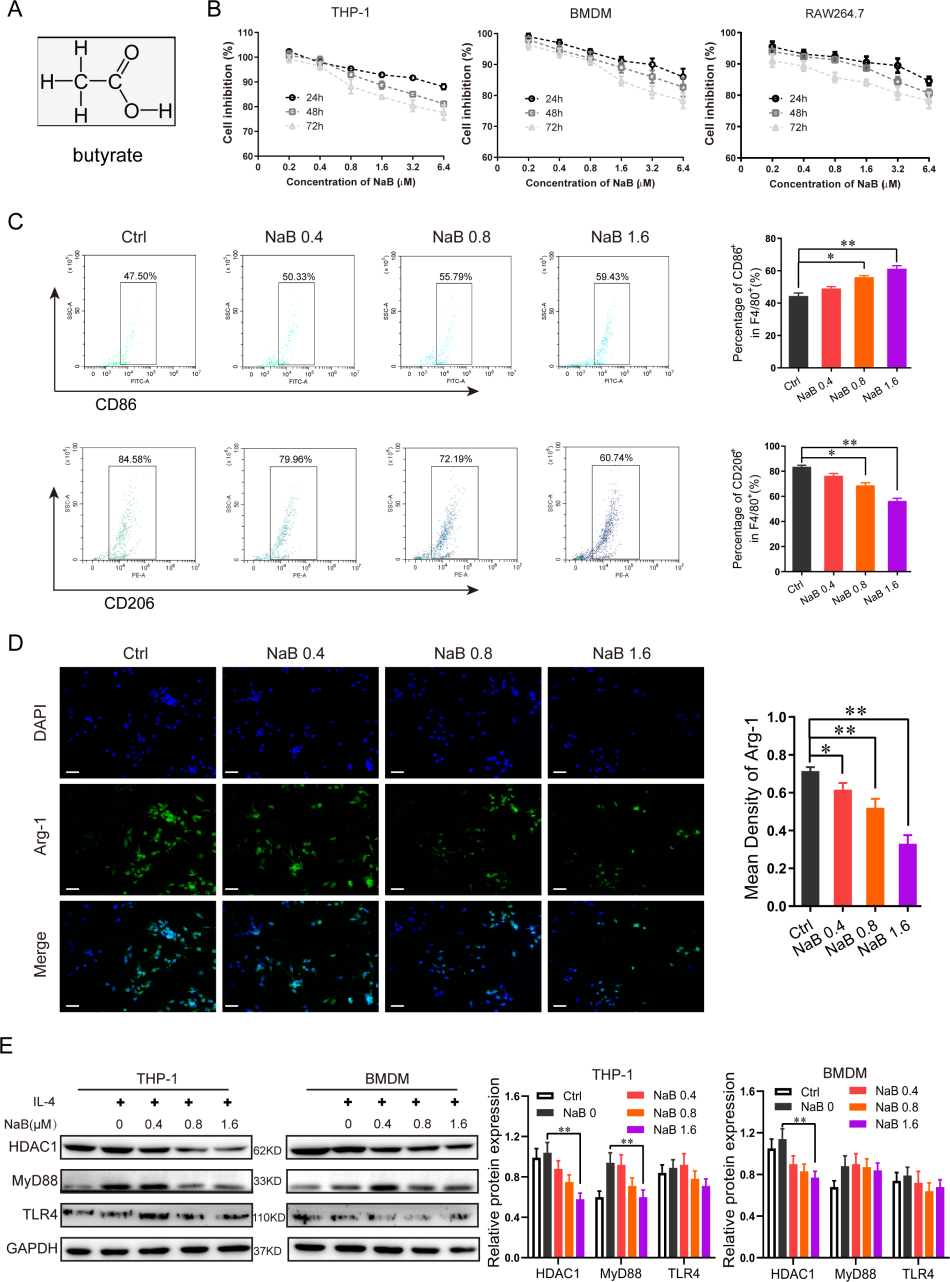
NaB inhibited the polarization of M2 macrophages through the HDAC/TLR4 signaling pathway *in vitro*. (**A**) Molecular formula of NaB. (**B**) Effect of NaB on macrophage viability, including RAW264.7, BMDM, and THP-1, for 24 h, 48 h, and 72 h. (**C**) The percentage of CD86^+^ and CD206^+^ in F4/80^+^ cells was detected by flow cytometry after treatment with different concentrations of NaB (0.4 mM, 0.8 mM, and 1.6 mM). The results are from one of two or three independent experiments. The data are presented as the mean ± SD (*n* = 5), with Welch’s correction, two-tailed *t*-test. **P < 0.01. (**D**) Immunofluorescence micrographs of Arg-1 (green) and DAPI (blue) expression in macrophages after treatment with different concentrations of NaB. Scale bar = 100 µm. (**E**) Relative protein expression levels of HDAC1, MyD88, and TLR4 determined by Western blot analysis in M2 macrophages treated with different concentrations of NaB. GAPDH as the loading control. The data are presented as the mean ± SD (*n* = 3), with Welch’s correction, two-tailed *t*-test. *P < 0.05, **P < 0.01.

As shown in Fig. 3A, NaB is a straight-chain saturated fatty acid with the chemical formula C_4_H_8_O_2_. In experimental systems, butyric acid is predominantly utilized as its sodium salt, sodium butyrate (NaB), due to superior stability and aqueous solubility. It serves as a practical source for delivering butyrate to investigate its biological functions, particularly histone deacetylase inhibition (HDACi) (37). By deacetylating critical nodes in the TLR4/MyD88 cascade, HDAC-dependent epigenetic mechanisms shape macrophage polarization trajectories (38). To investigate the impact of NaB on the polarization of macrophages toward the M2 phenotype, we used three different sources of macrophages (THP-1, RAW264.7 cell lines, and BMDMs) and treated them with varying concentrations of NaB. The cell viability assay results showed that NaB inhibited the growth of macrophages in a dose- and time-dependent manner (Fig. 3B). Consistent with prior findings (39), an NaB dose of up to 1.6 mM had a minimal impact on macrophage viability (Fig. 3B). We next analyzed the effect of NaB on the macrophage phenotype using flow cytometry (Fig. S1). We found that NaB increased the proportion of CD86^+^F4/80^+^ THP-1 cells relative to the control in a dose-dependent manner (Fig. 3C, top panel), while decreasing that of CD206^+^F4/80^+^ macrophages (Fig. 3C, bottom panel). Similar results were obtained from experiments with BMDMs (Fig. S2). Meanwhile, NaB decreased the expression of Arg-1 by macrophages in a dose-dependent manner following treatment with NaB (Fig. 3D). To explore the effect of NaB on the HDAC/TLR4/MyD88 pathway, THP-1 cells and BMDMs were induced to differentiate into the M2 phenotype using IL-4 (20 ng/mL), before being exposed to different concentrations of NaB. Western blotting indicated that NaB significantly reduced the expressions of HDAC1 and MyD88 in THP-1 cells and BMDMs in a dose-dependent manner (Fig. 3E). Although it is not showing a dose dependency in the expression of TLR4, a downward trend can still be seen in THP-1 cells and BMDMs (Fig. 3E). Collectively, these results suggest that NaB inhibits M2 macrophage polarization via the HDAC/TLR4/MyD88 pathway.

### NaB supplementation enhances the antitumor efficacy of PD-L1 blockade

**Fig 4:**
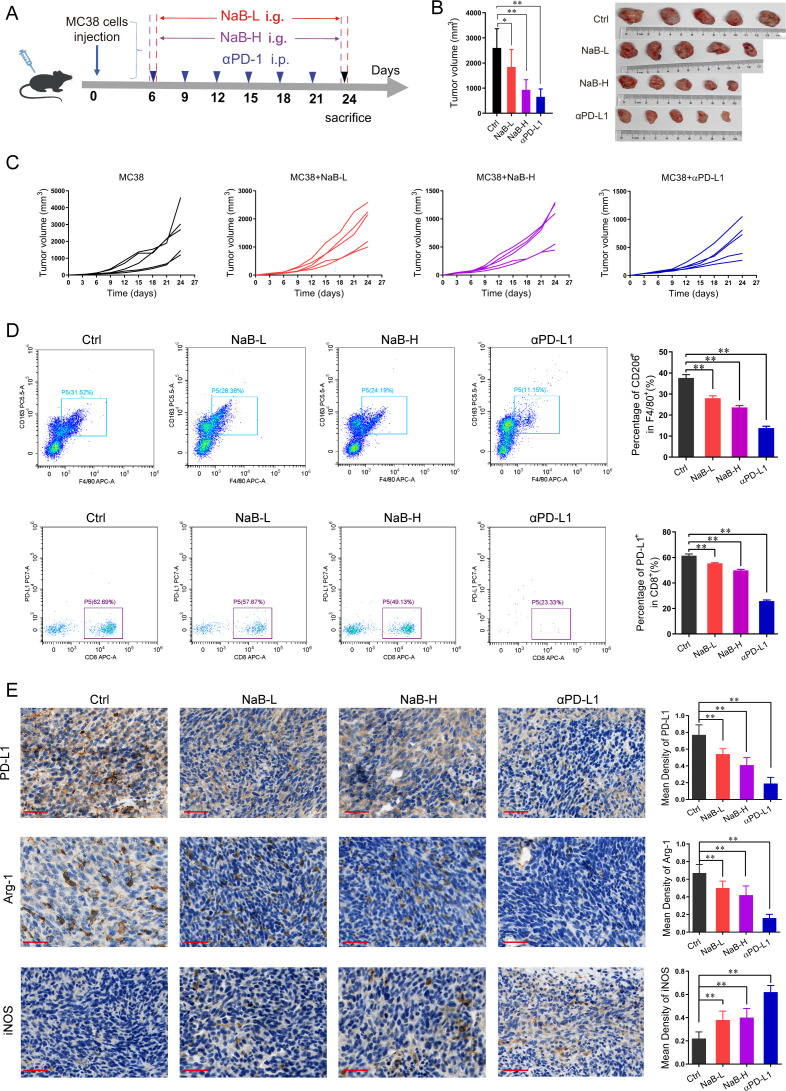
NaB inhibits the growth of CRC tumors by decreasing the expressions of PD-1 and PD-L1. (**A**) The diagram shows the experimental design and timeline. C57BL/6 J mice (*n* = 5 per group) were implanted with MC38 cells for 6 days and then treated with saline (0.2 mL i.g.), NaB-L (50 mg/kg, i.g.), and NaB-H (200 mg/kg, i.g.) every day till the mice were euthanized. The αPD-L1 group was treated with αPD-L1 (100 μg per mouse) once every 3 days for 18 days. Afterward, 24 days after the injection of MC38 cells, all of the mice were sacrificed. and tumor tissue samples were collected for flow cytometry and immunohistochemical staining. (**B**) Tumor volume in mice treated with either Ctrl (saline): shared control group, NaB-L, NaB-H, or αPD-L1 (left). Images of CRC tumors in mice after sacrifice (right). (**C**) Changes in tumor volume (V = L × W^2^/2) of mice were measured and recorded every 3 days during the disease process. (**D**) M2 macrophage-related CD206 + in F4/80^+^cells and PD-L1^+^ in CD8^+^cells were analyzed by flow cytometry (*n* = 5 per group). (**E**) PD-L1, Arg-1, and iNOS expression levels in the tumor tissues of the indicated groups were assessed using immunohistochemistry. Scale bar = 100 µm. Data represented mean ± SD. **P < 0.01.

Having shown that NaB inhibited macrophage polarization toward the M2 phenotype and regulated PD-L1 expression, we subcutaneously injected the MC-38 CRC cell line into our model mice (Fig. 4A). After 6 days, NaB-L, NaB-H, or αPD-L1 were administered to the CRC mice with established subcutaneous MC-38 tumors (Fig. 4A). As expected, both NaB and αPD-L1 significantly inhibited the growth of subcutaneously implanted tumors in a dose-dependent manner (Fig. 4B and C). Next, we used flow cytometry and immunohistochemistry to detect the expression of factors related to tumor immunotherapy. The results showed that both NaB and αPD-L1 reduced the infiltration of PD-L1-expressing cells into the subcutaneous tumors (Fig. 4D). Immunohistochemistry analysis of CRC mouse tumor tissue showed that NaB decreased the expressions of PD-L1, Arg-1, and iNOS in a concentration-dependent manner (Fig. 4E). Taken together, these findings reveal that NaB reduces the expression of PD-L1 on implanted CRC tumor cells.

### Effects of NaB in macrophage-depleted CRC-bearing mice

**Fig 5:**
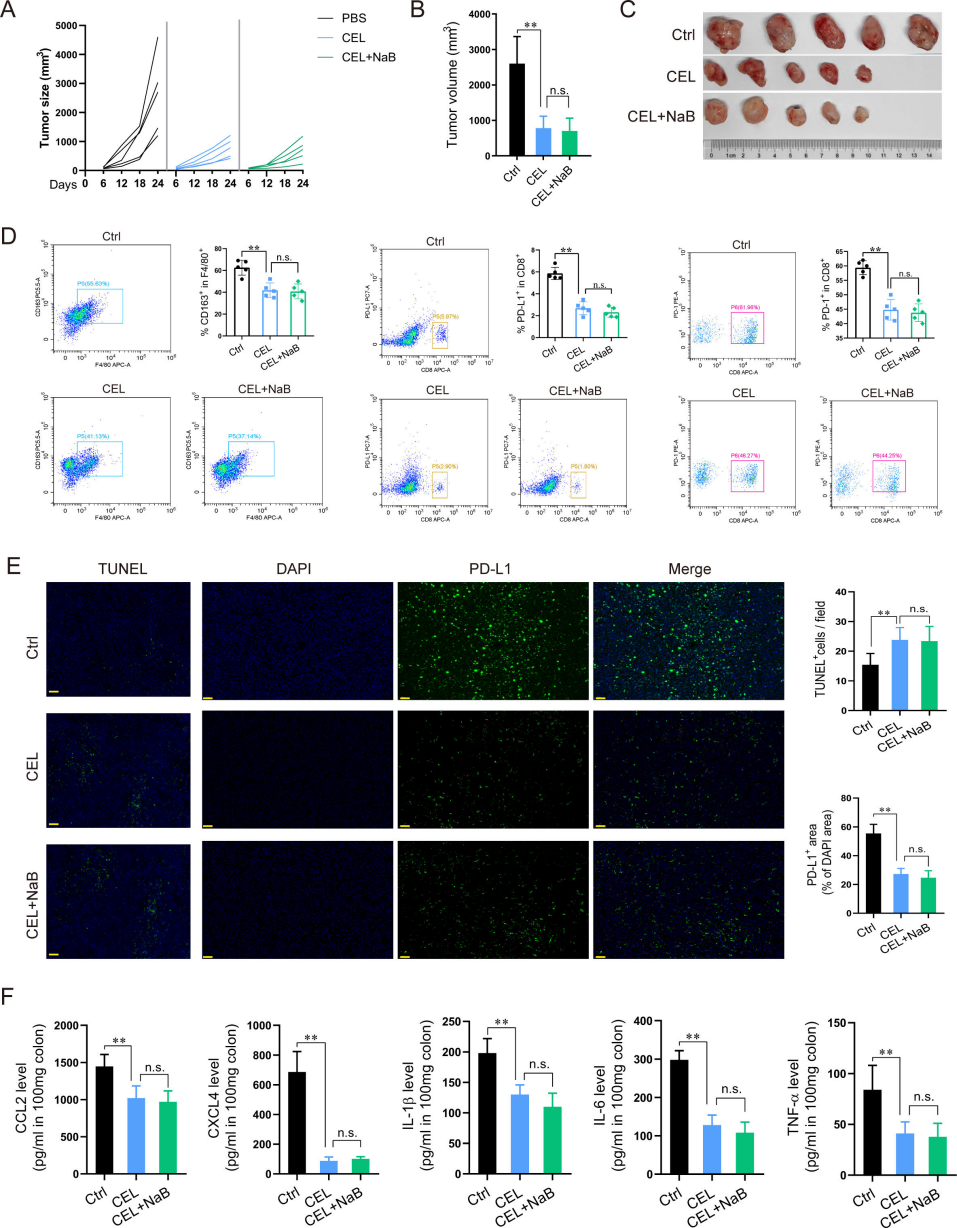
NaB has a weaker inhibitory effect on the CRC subcutaneous tumors in macrophage-depleted mice. (**A**) As the experimental design of Fig. 4A, C57BL/6 mice (*n* = 5 per group) were implanted with MC38 cells, followed by indicated therapy, including PBS (0.2 mL, ip), CEL (12.5 mg/kg, iv), or CEL plus NaB-M (100 mg/kg, i.g.) therapy (*n* = 5 per group). Changes in tumor size of mice were measured and recorded every 6 days in this process. (**B**) Tumor volume in the mice in each group at the end of the experiment (*n* = 5 per group). (**C**) Images of CRC tumors in mice at day 24 post-tumor implantation. Ctrl: shared control group. (**D**) M2 macrophages related CD163^+^ in F4/80^+^cells and PD-L1^+^ and PD-1^+^ in CD8^+^cells were analyzed by flow cytometry (*n* = 5 per group). (**E**) Expressions of PD-L1 (green) and DAPI (blue) of subcutaneous tumor tissue were detected by immunofluorescence (IF) staining, and representative images (scale bar = 50 µm) were performed. (**F**) CCL2, CXCL4, TNF-α, IL-6, and IL-1β levels in tumor tissues were evaluated using ELISA. The data are presented as the mean ± SD of at least three experiments. **P < 0.01, n.s., not significant.

To determine whether the inhibitory effects of NaB on subcutaneously implanted CRC tumors were macrophage-dependent, we used clodronate liposomes (CELs) to deplete macrophages *in vivo*. Briefly, the mice were treated with PBS, CELs, or CELs + NaB on days 6–24. We found that CEL treatment alone reduced the tumor size in mice; however, administering NaB did not decrease tumor size further (Fig. 5A through C). Moreover, while CEL administration significantly reduced the number of CD163^+^F4/80^+^ macrophages and PD-1^+^ or PD-L1^+^CD8^+^ T cells in the mice, the combination of CEL and NaB did not decrease their numbers further (Fig. 5D). Concurrently, the CEL and NaB combination did not decrease PD-L1 expression below that achieved with CEL alone (Fig. 5E). Our ELISA results indicated that CEL treatment decreased the levels of CCL2, CXCL4, IL-1β, IL-6, and TNF-α in mice; again, adding NaB did not further decrease the levels of these factors (Fig. 5F). These findings reveal that NaB inhibits tumor growth in a TAM-dependent manner.

### NaB inhibits tumor cell proliferation by regulating M2 macrophage activity *in vitro*

**Fig 6:**
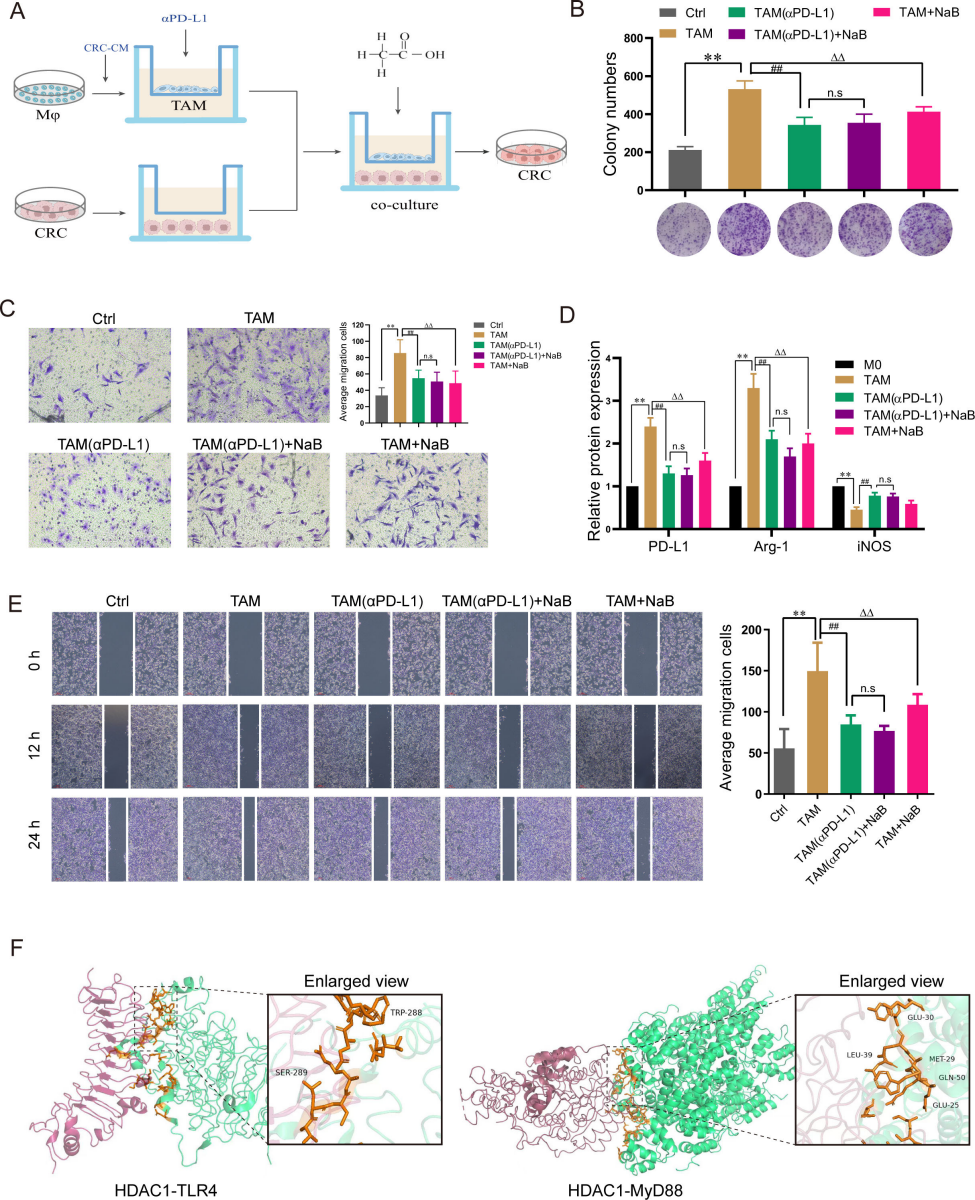
NaB inhibits the growth and migration of colorectal cancer cells through the PD-L1^+^ TAM *in vitro*. (**A**) Overview of study design. First, macrophages were induced to TAM by CRC-CM (Caco-2 Conditioned medium) and placed in the upper layer of the chamber. Subsequently, anti-PD-L1 (αPD-L1) and NaB (0.8 mM) were added separately, and CRC cells were placed in the lower layer to establish a coculture system. CRC cells were used for subsequent experiments. (**B**) Colony formation of CRC cells was treated with different interventions for 48 h. (**C**) Representative images and quantification of invaded cells (crystal violet staining) in different treatment groups. (**D**) Representative scratch experiment images at 0 h, 12 h, and 24 h and average migration of CRC cells. (**E**) mRNA expressions of PD-L1 and Arg-1 in different groups of macrophages. The data are presented as the mean ± SD of at least three experiments. **P < 0.01 vs control, ^##^*P* < 0.01 vs TAM. ^△△^*P* < 0.01 vs TAM. n.s., not significant. (**F**) Left: the molecular docking model of HDAC1-TLR4 was constructed using AutoDock; Right: the molecular docking model of HDAC1-MyD88 was constructed using AutoDock.

Having demonstrated that the inhibitory effects of NaB on CRC were TAM-dependent, we next cocultured CRC cells with macrophages that had been pretreated with CRC-CM; αPD-L1 and NaB were then added to this coculture system (Fig. 6A). We first used a colony formation assay to show that TAMs promoted the proliferation of CRC cells, while αPD-L1 and NaB reduced the proliferation ability of CRC cells, indicating that NaB inhibits the growth of CRC cells in the presence of TAMs (Fig. 6B). Next, we investigated the effect of NaB on the invasion of TAMs, and transwell assay results showed that NaB inhibited the invasiveness of TAMs (Fig. 6C). Consistent with the clone formation results, no significant differences in the invasive capacity of TAMs were observed between TAM (αPD-L1) and TAM (αPD-L1) +NaB groups, given that the NaB acts on macrophages via PD-L1. Furthermore, we used a wound healing assay to verify the migration ability of CRC cells at 0, 12, and 24 h after wound formation. We showed that αPD-L1 or NaB markedly inhibited the rate of wound closure (Fig. 6D). For further verification, qRT-PCR was performed using custom-designed primers to assess TAM-related gene expression in TAMs under different treatment conditions. We found that the expressions of PD-L1 and Arg-1 were increased in the TAM group and decreased in the TAM (αPD-L1) of the TAM^+^ NaB group, suggesting that the inhibition of PD-L1^+^ TAMs attenuated the tumor-promoting effects of TAMs (Fig. 6E). These results imply that NaB suppresses macrophage polarization by lowering their PD-L1 expression. As the prototypical HDAC antagonist, NaB was shown to significantly alleviate macrophage polarization by targeting TLR4/MyD88 signaling. As a method to verify the activation of TLR4 or MyD88 by HDAC, molecular dynamics simulation (MDS) was first used to mimic the potential interaction between the human HDAC1 domain and the TLR4 or MyD88 (Fig. 6F). The predicted binding energies of HDAC1 with TLR4 and MyD88 were 10.5 kcal/mol and 14.9 kcal/mol, respectively, suggesting that HDAC1 exhibits notable binding potential with both TLR4 and MyD88 (Fig. 6F). It indicates that HDAC1 likely plays a pivotal role in the activation of the TLR4/MyD88-dependent signaling cascade.

## DISCUSSION

**Fig 7:**
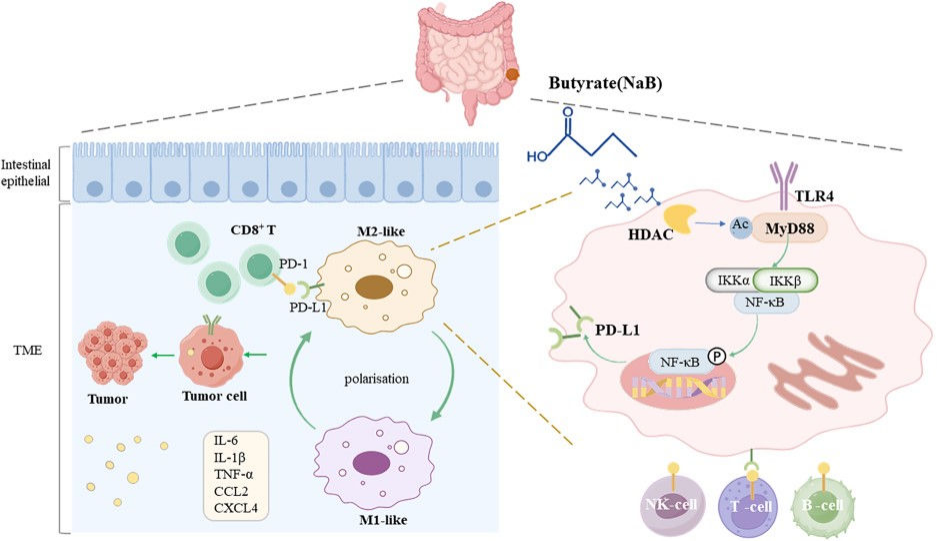
Diagram of the primary molecular mechanism through which NaB inhibits CRC. On the left, butyrate induces a phenotypic shift in macrophages from an M2-like to an M1-like phenotype and downregulates the expression of PD-L1^+^ macrophages. On the right, the TLR/MyD88 signaling pathway plays a crucial role in the modulation of macrophage polarization. Activated TLR4 binds to MyD88, stimulating the activation of IκB kinase (IKK) and subsequently leading to the activation of the NF-κB signaling pathway. This reversal facilitates the binding of tumor-associated antigens to T cells, leading to T cell activation through antigen-presenting cells. The activated T cells release various cytokines, which recruit macrophages to attack tumor cells, thereby exerting an anticancer effect.

The use of NaB is currently being explored as a potential strategy for the prevention and treatment of CRC. Recent studies have demonstrated that the abundance of NaB-producing bacteria is significantly reduced in the fecal matter of CRC patients (40, 41). Although NaB is being viewed as a potential anticancer drug candidate, the mechanisms underlying its ability to inhibit tumor progression are unclear (23). Our study demonstrated that NaB inhibited intestinal tumor development with a similar potency to that of aspirin in AOM-/DSS-induced CRC mice. Additionally, the antitumor effects of NaB were associated with the suppression of tumor cell proliferation and migration, an increase in the rate of tumor cell apoptosis, the modulation of the gut microbiota, and the inhibition of the HDAC/TLR4/MyD88 signaling pathway (Fig. 7).

Recent findings have shown that intestinal probiotics induce antitumor effects *in vitro* (using CRC cell lines) and *in vivo* (using chemically induced CRC mouse models) by stimulating SCFA production (42). Although SCFAs have long been recognized for their effectiveness in CRC treatment (43), NaB has only recently entered the research arena. By promoting CD8^+^ T-cell effector functions (IFN-γ/TNF-α secretion and granzyme B release), NaB synergizes with anti-PD-1 therapy (18). Simultaneously, it inhibits IFN-γ-triggered PD-L1 upregulation in tumor cells through STAT1 acetylation, thereby reversing immunosuppression (37). Our study found that NaB significantly reduced tumor load, inhibited tumor progression, and alleviated histological abnormalities in AOM-/DSS-induced CRC mice. The ability of NaB to inhibit the proliferation and promote the apoptosis of intestinal tumor cells was observed in both *in vivo* and *in vitro* experiments.

PD-L1 expression occurs in both tumor cells and infiltrating immune cells, with distinct predictive values for immunotherapy response. Sensitivity to PD-1/PD-L1 inhibitors is contingent upon the integrated immune microenvironment (e.g., CD8^+^ T cell abundance, exhaustion markers, and tumor mutational burden). It has been reported that the upregulation of PD-L1 on tumor cells recruits macrophages and drives tumor immune escape, thereby promoting CRC growth (44). Furthermore, single-cell transcriptomics enables identification of rare PD-L1^+^CD8^+^ subsets and their transcriptional profiles, but functional validation through flow cytometry or functional assays remains essential. Our study supports this hypothesis and justifies its further exploration. We focused on tolerogenic PD-L1-expressing CD8^+^ T cells and M2 macrophages differentially localized in the colonic tissues of CRC mice. Our findings indicate that NaB inhibited PD-L1 expression on tumor cells while suppressing macrophage polarization toward the M2 phenotype.

TAMs play a key role in tumor immune evasion. While M1-like TAMs induce antitumor immune responses by stimulating T cells, the M2-like TAMs promote tumor growth and metastasis by inhibiting T cell expression. TAMs are polarized to the M2-like phenotype by multiple mediators, including M-CSF and IL-10 (45). In the present study, we showed that NaB treatment inhibited the polarization of three different sources of macrophages toward the M2 phenotype. The dysregulation of the TLR signaling network is a critical event in CRC progression (46). Previous studies have confirmed that TLR4 is a key molecule in the inflammatory response and that the activation of the TLR4 signaling pathway can stimulate the M2 polarization of TAMs (47, 48). Consistent with these findings, we found that NaB, as an HDACi, inhibited the expressions of HDAC1, TLR4, and MyD88 on TAMs. The results suggest that the inhibition of TAM M2 polarization by NaB may be related to its ability to block the binding of HDAC1 to TLR4/MyD88, thereby inhibiting this pathway.

Mechanistically, the interaction between PD-1 and PD-L1 dampens the antitumor activity of cytotoxic T cells, leading to tumor immune escape. We found that NaB exhibited similar antitumor effects to PD-L1 inhibitors; both agents reduced tumor load, decreased PD-L1 expression on tumor cells, inhibited M2-like macrophage polarization, and promoted M1-like macrophage polarization.

Given that TAMs express PD-1, blocking the PD-1/PD-L1 has been shown to increase their antitumor potential (45). To determine whether the inhibitory effects of NaB on subcutaneously implanted CRC tumors were macrophage-dependent, we performed experiments using a macrophage-depleted CRC-bearing mouse model. We found that TAM depletion significantly reduced the expression of M2-type macrophage markers, PD-L1, and related inflammatory factors in tumor cells. Crucially, NaB treatment ceased to affect the levels of these indicators or the size/volume of the tumor both *in vivo* and *in vitro* following TAM depletion. Conversely, NaB or αPD-L1 inhibited the proliferation, invasion, and migration of CRC cells in a coculture system with TAMs; moreover, the combination of NaB and αPD-L1 exerted the strongest antitumor effect. These results suggest that NaB inhibited CRC growth by modulating TAMs and that the combination of PD-L1 inhibitors and NaB represents a promising therapeutic option for CRC in the future.

### Conclusions

In summary, our study explored the role of NaB in CRC development both *in vivo* and *in vitro*. The results suggest that NaB elicits its antitumor effect by inhibiting M2-like macrophage polarization and reducing PD-L1 expression on tumor cells. The specific mechanism of NaB may be related to its regulation of the HDAC/TLR4/MyD88 signaling pathway. Moreover, given that NaB improves the efficacy of PD-L1 inhibitors, this combination could be exploited in the prevention and treatment of CRC in the future.

## Data Availability

The sequenced data reported in this article have been submitted to the NCBI Sequence Read Archive (SRA) under accession no. PRJNA954981. Data are available on reasonable request. All data relevant to the study are included in the article and its supplemental material.

## SUPPLEMENTAL MATERIAL

Supplemental material msystems.00692-25-s0001.docx Tables S1 to S3; Fig. S1 to S3.

ASM does not own the copyrights to Supplemental Material that may be linked to, or accessed through, an article. The authors have granted ASM a non-exclusive, world-wide license to publish the Supplemental Material files. Please contact the corresponding author directly for reuse.
