# Supplementary material for: Sodium butyrate inhibits colorectal cancer development by reducing M2 macrophage polarization and PD-L1 expression
Source: mSystems. 2025 Nov 18;10(12):e00692-25. doi: 10.1128/msystems.00692-25 (PMC12710303; doi:10.1128/msystems.00692-25)
Supplement: Supplemental material — Tables S1 to S3; Fig. S1 to S3. [file msystems.00692-25-s0001.docx]

**Supplementary Data**

**Supplementary Table**

**Supplementary Table S1:** **Histopathological Grading Criteria for Colorectal Adenomas**

| Histological Parameter | Grading Criteria | Score |
| --- | --- | --- |
| Epithelial Architecture Disruption | none | 0 |
|  | <25% of the field involved) | 1 |
|  | Moderate distortion (25-50% involved) | 2 |
|  | Severe distortion (50-75% involved) | 3 |
|  | Complete loss of structure (>75% involved) | 4 |
| Goblet Cell Depletion | Normal density and distribution | 0 |
|  | Focal loss (<25% reduction) | 1 |
|  | Partial loss (25–50% reduction) | 2 |
|  | Extensive loss (50–75% reduction) | 3 |
|  | Absent (>75% reduction) | 4 |
| Crypt Damage/Absence | Intact crypts | 0 |
|  | <25% crypts distorted or absent | 1 |
|  | 25–50% crypts distorted or absent | 2 |
|  | 50–75% crypts distorted or absent | 3 |
|  | >75% crypts destroyed or absent | 4 |
| Lamina Propria Inflammatory Cell Infiltration | Rare lymphocytes/plasma cells | 0 |
|  | Mild increase (<25% of lamina propria) | 1 |
|  | Moderate increase (25–50%) | 2 |
|  | Marked increase (50–75%) | 3 |
|  | Diffuse infiltration (>75%) | 4 |

Total Inflammation Score: Sum of scores for all 4 parameters (Range: 0–16).

**Supplementary Table S2. PCR primers**

| **Genes** | **Species** | **Forward primer** | **Reverse primer** |
| --- | --- | --- | --- |
| PD-L1 | Human | GGCATTTGCTGAACGCCC | TTGGAGGATGTGCCAGAGGT |
| Arg-1 | Human | ACGGAAGAATCAGCCTGGTG | GTCCACGTCTCTCAAGCCAA |
| iNOS | Human | TCCAAGGTATCCTGGAGCGA | CAGGGACGGGAACTCCTCTA |
| HDAC1 | Human | GTTCTGTGGCAAGTGCTGTG | CACACTTGGCGTGTCCTTTG |
| MyD88 | Human | ACTTGGAGATCCGGCAACTG | ATCCGGCGGCACCAATG |
| TLR4 | Human | CCAAGAACCTGGACCTGAGC | ACCCGCAAGTCTGTGCAATA |

**Supplementary Table S3. Histopathologic analysis of neoplastic lesions and the degree of dysplasia**

| **Group** | **Normal/mouse number** | **low-grade dysplasia/mouse number** | **high-grade dysplasia/mouse number** | **adenocarcinoma/mouse number** |
| --- | --- | --- | --- | --- |
| Control | 0 | 1/8 | 2/8 | 5/8 |
| NaB | 1/8 | 3/8 | 1/8 | 3/8 |
| Aspirin | 1/8 | 2/8 | 2/8 | 3/8 |

Histopathologic analysis of neoplastic lesions and the degree of dysplasia were assessed according to standard criteria and classification of adenomas of the colon. tubular adenoma with high-grade dysplasia characterized; low grade adenocarcinomas with focal submucosal invasion [1].

**Supplementary Figure**


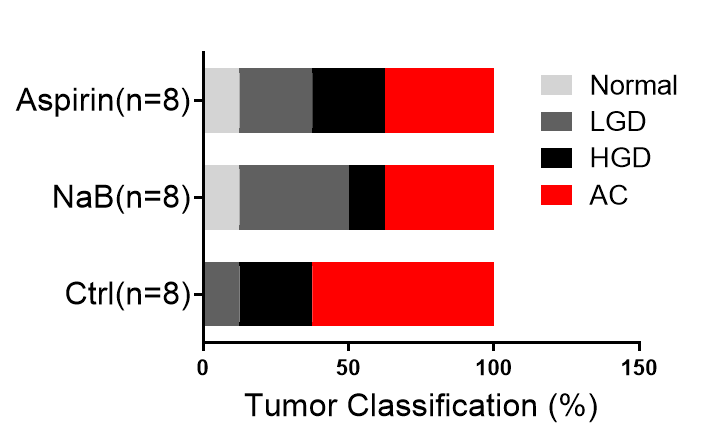


**Supplementary Figure S1 Histopathologic analysis of neoplastic lesions and the degree of dysplasia**

The incidence of low-grade dysplasia (LGD), high-grade dysplasia (HGD) and adenocarcinoma (AC) in the AOM/DSS CRC mouse with different treatment.


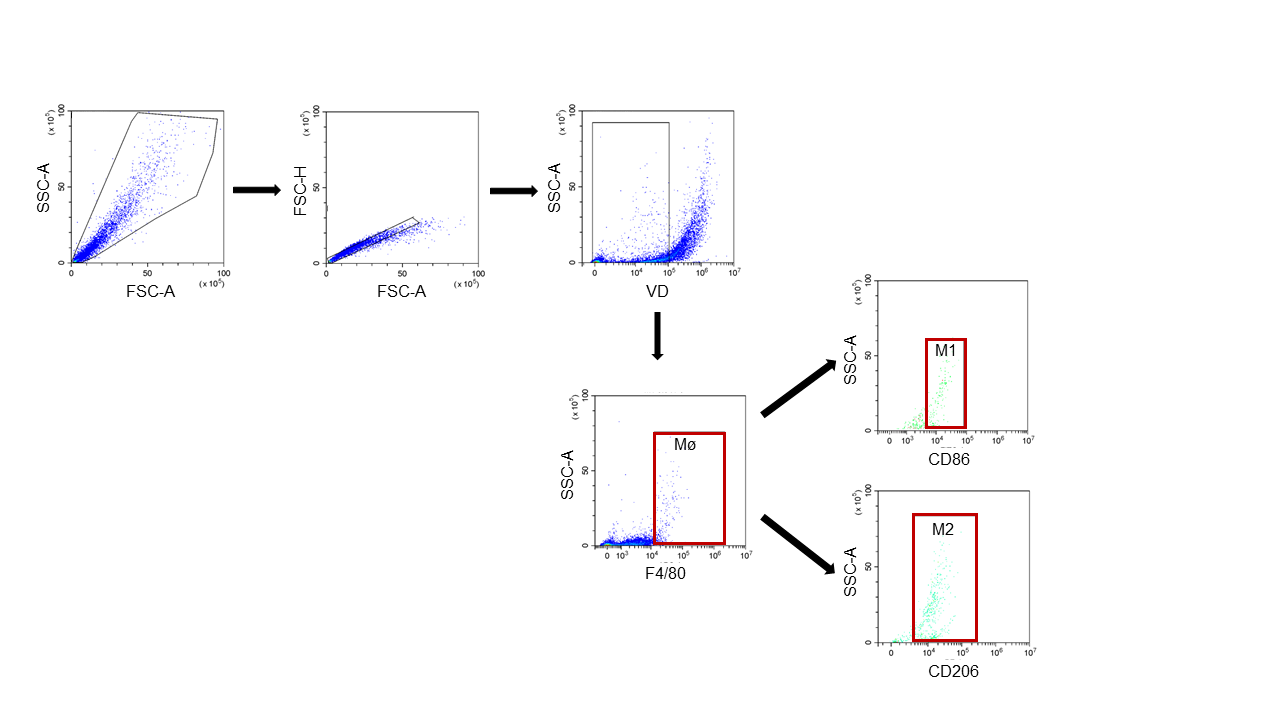


**Supplementary Figure S2** **Gating strategy for FITCs and PEs.**

Flow cytometry plots show the gating strategy for subtypes in pre-gated CD86^+^ cells and CD206^+^ cells from THP-1. Shown are representative results from three independent experiments.

| 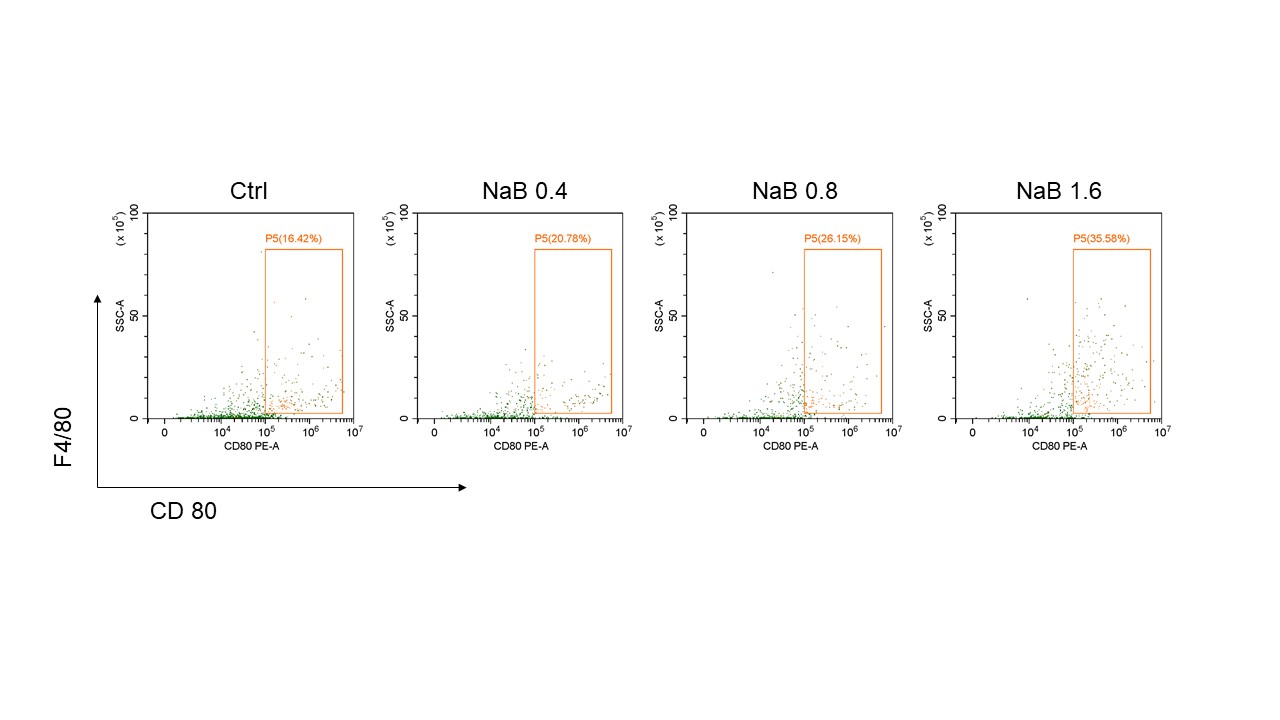 | 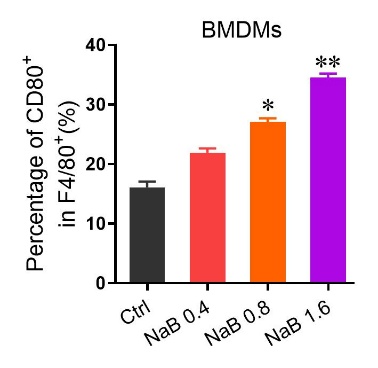 |
| --- | --- |
| 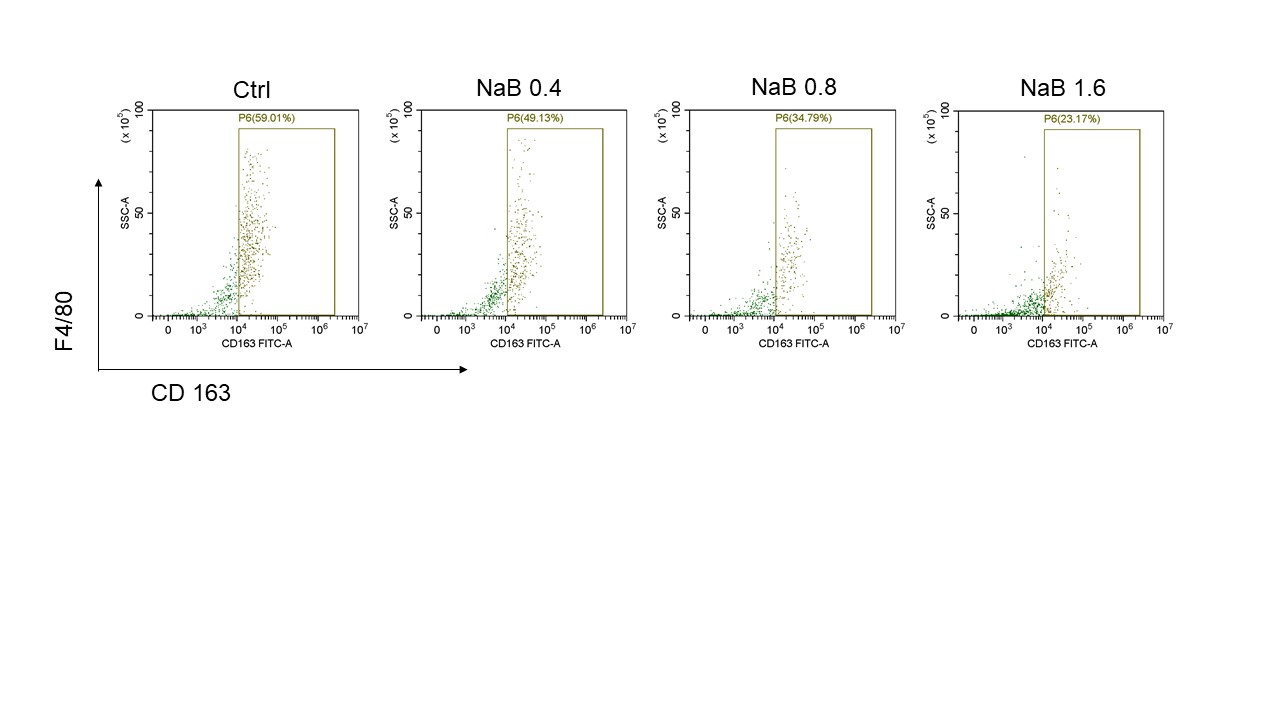 | 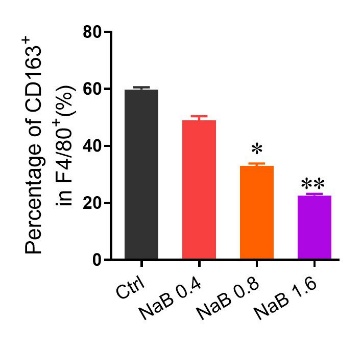 |

**Supplementary Figure S3** **The phenotype of M1 and M2 macrophage in BMDMs was examined.**

The percentage of CD80^+^ and CD206^+^ in F4/80^+^ were detected by flow cytometry after treatment with different concentration of NaB (0.4mM, 0.8mM and 1.6mM). The results are from one of two or three independent experiments. The data are presented as the mean ± SD (n=5), with Welch’s correction, two-tailed t-test. **P* < 0.05, ***P* < 0.01

**Reference**

1. Poutahidis T, Rao VP, Olipitz W, Taylor CL, Jackson EA, Levkovich T, Lee CW, Fox JG, Ge Z, Erdman SE. CD4+ lymphocytes modulate prostate cancer progression in mice. *Int J Cancer*. ***2009***;125(4):868-78.
